# Plasma Homocysteine and Autonomic Nervous Dysfunction: Association and Clinical Relevance in OSAS

**DOI:** 10.1155/2020/4378505

**Published:** 2020-07-08

**Authors:** Lei Liu, Qiansheng Wu, Hong Yan, Xilong Zheng, Qiang Zhou

**Affiliations:** ^1^Division of Cardiology, Department of Internal Medicine, Tongji Hospital, Tongji Medical College, Huazhong University of Science and Technology, Wuhan, China; ^2^Department of Biochemistry and Molecular Biology, Cumming School of Medicine, Libin Cardiovascular Institute of Alberta, University of Calgary, Alberta, Canada; ^3^Division of Cardiothoracic and Vascular Surgery, Tongji Hospital, Tongji Medical College, Huazhong University of Science and Technology, Wuhan, China

## Abstract

**Objective:**

Elevated plasma homocysteine (Hcy) is an independent risk factor for cardiovascular diseases, but the precise mechanism of Hcy in cardiovascular disease remains elusive. This study is aimed at evaluating the association between Hcy levels and autonomic nervous system and at investigating their clinical relevance in obstructive sleep apnea syndrome (OSAS).

**Methods:**

A total of 191 subjects with OSAS were enrolled for this cross-sectional study. Heart rate variability (HRV) represents the status of the autonomic nervous system and is a well-known index that allows studying the autonomic modulation. HRV and polysomnography parameters were collected based on Holter monitors and polysomnography system. The software computed all the basic HRV parameters including SDANN, SDNN and pNN50. Correlation analyses between Hcy and HRV parameters and echocardiographic parameters were performed.

**Results:**

Compared with the mild-moderate OSAS group, the prevalence of male and smoking and Hcy levels were considerably higher in the severe OSAS group (*P* = 0.01, *P* = 0.02, and *P* = 0.01, respectively). Also, there were significant linear relationships between Hcy quartiles with the proportion of severe OSAS (*P* = 0.01 for the trend). Interesting, there is a negative linear correlation between SDANN and Hcy quartiles (*P* = 0.02 for the trend). Spearman's correlation analysis showed a significant negative correlation between SDANN and Hcy levels (*r* = −0.17, *P* = 0.02). Interestingly, the relationship of it remains significant after adjustment for clinical covariates (*r* = −0.15, *P* = 0.04). However, echocardiographic parameters were not significantly correlated with Hcy or HRV parameters (all *P* > 0.05).

**Conclusions:**

Elevated plasma Hcy level is linearly correlated with cardiac autonomic nervous function disorders in patients with OSAS.

## 1. Introduction

Accumulating evidence suggests that abnormally elevated level of homocysteine (Hcy) is prevalent and an independent risk factor for various cardiovascular diseases and metabolic diseases [1, 2]. Hcy is a sulfur-containing nonconstitutive amino acid that is derived from the essential amino acid methionine, which is synthesized during metabolic conversion of methionine to cysteine in the liver [3]. Both epidemiologic and longitudinal clinical investigations have demonstrated that Hcy is affected by health-related behaviors, including diet, smoking, and sedentary lifestyle [4]. Meanwhile, it is well known that plasma Hcy levels can be altered by genetic factors. Although links have been established between elevated Hcy level and increased risk for cardiovascular events, the precise mechanism of Hcy in cardiovascular disease remains elusive [5]. The impact of Hcy on cardiovascular disease is best documented in terms of its association with increased platelet aggregation, proliferation of vascular smooth muscle, accelerated atherosclerosis, inflammatory monocyte differentiation, reduced the generation of nitric oxide, and left ventricular hypertrophy [6–8]. More recent data, however, suggests that elevated Hcy provokes an activation of the sympathetic system, and this fact contributes to cardiovascular diseases [9].

Obstructive sleep apnea syndrome (OSAS) has been increasingly recognized as a complex and heterogeneous condition, which is characterized by tumultuous snoring, recurrent occurrences of upper airway obstruction during sleep, and nocturnal hypoxemia [10]. Patients with OSAS usually present excessive daytime sleepiness, the greater risk of coronary artery disease, heart attack, heart failure, and stroke [11]. There is accumulating evidence proving that OSAS has a closed relationship with the dysfunction of the sympathetic nervous system [12]. Although experimental evidence suggests that the cardiovascular system may be susceptible to Hcy-induced injury, it is unclear whether the elevated level of Hcy is independently associated with the status of the autonomic nervous system. Therefore, the assessment of association between Hcy and the autonomic nervous system in OSAS patients is an important step in clinical settings.

Heart rate variability (HRV) reflects the status of the autonomic nervous system and is a well-known index that allows studying the autonomic modulation of the cardiac sympathovagal balance [13, 14]. Emerging studies have demonstrated that HRV is mainly associated with a sympathetic tone likely reflecting the severity of OSAS [15]. Excessive activity of the sympathetic link of the autonomic nervous system is one of the potential causes leading to cardiovascular remodeling [16]. Therefore, additional studies are needed to establish the relationship between HRV and functional and structural abnormalities of the myocardium.

Thus, the objective of this study was to explore the associations between Hcy and HRV in OSAS. We also investigated the relationships between HRV and the echocardiographic parameters in the subjects.

## 2. Materials and Methods

### 2.1. Study Design and Subjects

A total of 191 untreated OSAS patients were consecutively recruited from people who admitted to the Tongji Hospital in Wuhan (Hubei, China) between December 2015 and February 2018. Each participant underwent eight-hour nocturnal monitoring using polysomnography in bed. Polysomnographic recordings were done with Embla N7000 system (Medcare Embla, Reykjavik, Iceland) using Somnologica version 3.3.1 software (Medcare Embla, Reykjavik, Iceland). Airflow was continuously measured by a thermistor and a nasal pressure cannula. The respiratory movements were monitored using the respiratory inductive plethysmographic belts around the chest and abdomen. Oxygen saturation was measured by a pulse oximeter sensor which was put on the left second finger. Electrocardiographic signals acquired by the twenty-four-hour electrocardiogram (ECG) Holter monitoring (DMS 300-4, HolterReader, Producer DMS, Nevada, USA).

The study protocol was approved by the Ethical Committee of Tongji Hospital. Written informed consent was obtained from each participant before inclusion in this study. This study was conducted in accordance with the principles expressed in the Declaration of Helsinki.

### 2.2. Anthropometric and Biochemical Measurements

Hypopnea was defined as a reduction of airflow by 50-80% for at least 10 sec associated with either oxygen desaturation of at least 4% or arousals. Apnea was defined as an air flow reduction 80% or more for at least 10 seconds. Apnea-hypopnea index (AHI) was calculated by dividing the total number of apneas and hypopneas by the number of hours of sleep. The subjects were categorized into two groups, the mild-moderate OSAS group (*n* = 120) and the severe OSAS group (*n* = 71). The former was defined as the subjects with the AHI greater than 5/hour but less than 30/hour, and the later with the AHI equal to or greater than 30/hour.

The average HRV parameters of all segments were calculated as the monitored HRV parameters. The software computed all of the basic HRV parameters using the HRV analysis module. The HRV time domain variables were SDNN, SDANN, and pNN50. SDNN is the standard deviation of normal-to-normal intervals. SDANN is the standard deviation of the averages of normal-to-normal intervals in all 5 min segments. pNN50 is the NN50 count divided by the total number of all normal-to-normal intervals. NN50 count means the number of pairs of adjacent normal-to-normal intervals differing by more than 50 ms in the entire analysis interval.

A standard echocardiographic examination was performed in all participants (GE Vingmed Vivid 7 or Vivid 9, Horten, Norway). Left ventricular end-diastolic dimension (LVEDD) was measured using M-mode in the parasternal left ventricular (LV) long axis view. Left ventricular biplane Simpson method ejection fraction (EF) was measured in apical 4- and 2-chamber views. The parameters of LV diastolic function were measured by recording transmitral flow velocity using Doppler echocardiography. The peak velocities of early (E velocity) and late (A velocity) transmitral flow were measured, and the E/A ratio was calculated.

Information on sex, age, ethnicity, and medical history was obtained through self-administered questionnaires. Blood samples were collected after an overnight fast in the morning to avoid potential confounding influences. Serum and plasma were stored in aliquots without preservatives at −80°C. Serum and plasma parameters were determined at the Department of Medical and Chemical Laboratory Diagnostics of Tongji Hospital according to routine procedures.

### 2.3. Statistical Analysis

Descriptive and experimental measures are expressed as the means ± SD or percentages as indicated. The distribution of quantifiable variables was tested for normality using a one-sample Kolmogorov–Smirnov test. Unpaired Student's *t*-test for normal distribution and Mann–Whitney *U* tests for asymmetric distribution were used to analyze differences in continuous variables. Categorical values were compared by the *χ*^2^ test or Fisher's test when appropriate. Comparisons between groups were made using an independent *t*-test, analysis of variance (ANOVA), or Wilcoxon's rank sum test. Relations between variables were determined by Spearman's correlation coefficients analysis. Statistical and association analyses were performed using SPSS 15.0 (SPSS Inc., Chicago, Illinois, USA). All tests were two-sided, and *P* values less than 0.05 were considered statistically significant.

## 3. Results

### 3.1. Clinical Characteristics

A total of 191 individuals were evaluated. They were divided into two groups, the mild-moderate OSAS group (*n* = 120) and the severe OSAS group (*n* = 71). The clinical characteristics of the two groups are shown in Table 1. We found notable differences regarding gender and smoking between the two groups. The severe OSAS group had significantly higher male and smokers as expected. Of note, the severe OSAS group had significantly higher levels of Hcy than the mild-moderate OSAS group (17.4 ± 6.9 vs. 14.9 ± 6.0 *μ*mol/L, *P* = 0.01). There were no significant differences in age, BMI, UA, FBG, TG, TC, LDL, and HDL between the two groups. Meanwhile, no differences were observed for the *E*/*A* ratio, EF (%), and LVEDD between the groups.

### 3.2. Correlation Analysis according to Hcy Quartiles

In stratified analysis, we analyzed the proportion of severe OSAS, HRV parameters, and myocardium parameters according to Hcy quartiles. The respective ranges for Hcy (*μ*mol/L) quartiles for all participants were as follows: Q1 (<11.3), Q2 (11.3-14.3), Q3 (14.4-18.3), and Q4 (>18.3). As presented in Table 2, higher Hcy levels were associated with a higher proportion of severe OSAS (*P* = 0.04). With increasing Hcy, there were significant linear relationships between Hcy quartiles and severe OSAS (*P* = 0.01 for the trend). Interestingly, there is a negative linear correlation between SDANN and Hcy quartiles (*P* = 0.02 for the trend). However, the linear correlation between the other HRV parameters (SDNN and pNN50) and Hcy quartiles was not significant (all *P* value for the trend > 0.05). No differences were observed for HRV parameters (SDANN, SDNN, and pNN50) among Hcy quartile groups (all *P* value > 0.05). Also, myocardium parameters (*E*/*A* ratio, EF (%), and LVEDD) were not significantly different among Hcy quartile groups (all *P* value > 0.05). Correlation analyses were according to Hcy levels.

**Figure 1:**
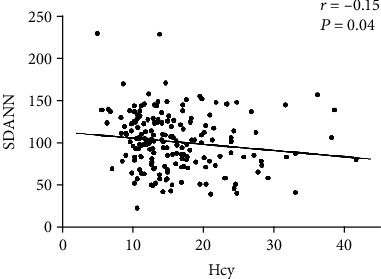
The correlation between plasma Hcy levels and SDANN in OSAS subjects.

We next studied correlations between Hcy levels and HRV parameters and myocardium parameters in all subjects. Univariable and multivariable analyses were carried out as presented in Table 3. Of note, there was a significant negative correlation between SDANN and Hcy levels (*r* = −0.17, *P* = 0.02). Interestingly, the relationship of it remains significant after adjustment for clinical covariates (*r* = −0.15, *P* = 0.04) (Figure 1). Furthermore, SDNN tended to marginally correlated negatively with Hcy levels (*r* = −0.15, *P* = 0.03). However, this association disappeared after adjustment for clinical covariates (*r* = −0.10, *P* = 0.16). In contrast, no significant correlation was found between pNN50 and Hcy levels (*P* > 0.05). There was no significant correlation between myocardium parameters (*E*/*A* ratio, EF (%), and LVEDD) and Hcy levels (all *P* > 0.05).

### 3.3. Correlation Analyses between HRV and Myocardium Parameters

Furthermore, we then conducted analysis for the correlations between HRV parameters (SDANN, SDNN, and pNN50) and myocardium parameters (*E*/*A* ratio, EF (%), and LVEDD). As shown in Table 4, there was no statistically significant correlation of HRV parameters (SDANN, SDNN, and pNN50) with myocardium parameters (*E*/*A* ratio, EF (%), and LVEDD) (all *P* > 0.05).

## 4. Discussion

The present study cross-sectionally examined the relationship of plasma Hcy to the autonomic nervous system in OSAS individuals. Evidence exists in the literature that Hcy is associated with diverse parameters of subclinical cardiovascular diseases such as atherosclerosis, metabolic syndrome, and endothelial dysfunction [1, 17]. Our findings extend these observations by demonstrating that elevated Hcy levels are closely associated with dysfunction of the autonomic nervous system independent of potential confounders among individuals of OSAS.

In accordance with previous evidence, our study has confirmed that severe OSAS patients showed higher plasma Hcy levels in comparison to mild-moderate. With increasing Hcy, there were significant linear relationships between Hcy quartiles and severe OSAS. Similarly, a previous study demonstrated an association between homocysteine and OSAS severity in males [18]. Consistently, subjects with acute coronary syndrome in the moderate-severe OSAS group had a higher homocysteine compared with those with no or mild OSAS [19]. Although the exact mechanism remains incompletely clarified, another study has also demonstrated that the severity of OSAS is significantly associated with an elevated level of homocysteine in ischemic stroke patients [20]. Thus, future studies will be needed to clarify the role of Hcy in the pathogenesis of OSAS.

It is well known that cardiac function is under tight control of the autonomic nervous system with its parasympathetic and sympathetic branches. During the past decade, several studies have demonstrated an interaction between the autonomic system and cardiovascular diseases [21, 22]. In agreement with its central role in cardiac physiology, targeting effector pathways of the autonomic nervous system have evolved as a cornerstone of medical therapy for a number of cardiac conditions.

Accumulating evidence found that hyperhomocysteine might induce the dysfunction of the renin-angiotensin system during vascular remodeling [23, 24]. Of note, a previous study has demonstrated that hyperhomocysteine directly interacts and activates the renin-angiotensin-aldosterone system (RAAS) to aggravate dysfunction of the autonomic nervous system [25]. Studies using genetic- and diet-induced animal models of hyperhomocysteine show that physiopathological features of hyperhomocysteine are similar to some physiopathological features found in sympathetic overactivity in the cardiovascular system [26].

However, it is unclear whether elevated Hcy levels are closely associated with autonomic dysfunction in clinical setting. Accordingly, we performed the current cross-sectionally study to clarify this important issue. Consistent with previous studies of hyperhomocysteinemia and cardiovascular autonomic system [9, 27], we found that elevated Hcy levels are significantly associated with the dysfunction of the autonomic nervous system in OSAS patients. Although no evidence of a causal effect of hyperhomocysteinemia on the autonomic nervous system was found by Mendelian randomization analysis, future studies are needed to investigate the implication of hyperhomocysteinemia in the autonomic nervous system.

In fact, there is accumulating literature reporting that hyperhomocysteinemia may provoke an activation of the sympathetic system, and this fact contributes to vascular and consequently organ damage [9]. Very recently, clinical evidence has demonstrated that elevated homocysteine concentrations decrease the antihypertensive effect of angiotensin-converting enzyme inhibitors in hypertensive patients [28]. However, the role of Hcy in the activation of sympathetic activity remains need to be determined in the future.

Subsequently, we evaluated the relationship between Hcy and echocardiographic parameters in this study. It has previously been reported that the elevated Hcy level is associated with reduced regional left ventricular systolic function in asymptomatic population [7]. We could not find significant correlation between Hcy and functional and structural abnormalities of the myocardium in the current study. However, importantly, our results do not rule out a potential association of Hcy and myocardium parameters. One explanation for the observed heterogeneity might be the small sample size in our study. Multiple linear analyses were performed in a total of 1178 subjects in the former study. On the other hand, myocardium parameters were calculated from magnetic resonance imaging in a previous study. In contrast, echocardiographic examination was performed in all participants to calculate myocardium parameters in the present study. Thus, the relationship between Hcy and functional and structural abnormalities of the myocardium needs to be clarified in future studies.

Meanwhile, many studies demonstrated significant effects of gender and age on the autonomic nervous system [29]. In accordance with these results, we found notable differences regarding gender between the mild-moderate OSAS group and the severe OSAS group in this study. It has also been found that menopause is an important factor for autonomic dysfunction [30]. In addition, age-related changes in autonomic dysfunction are also well known. The difference of autonomic dysfunction indices in pre- and postmenopausal women has been reported [31]. However, the influence of hormonal status in women before and after menopause needs further investigation. Therefore, the findings need to be interpreted with caution given the potential confounding effects of these factors.

## 5. Conclusion

This study demonstrated that the elevated plasma Hcy level was significantly and independently associated with HRV parameters in patients with OSAS. These findings support the notion that the increased plasma Hcy level is linearly correlated with cardiac autonomic nervous function disorders in patients with OSAS. However, further studies on a larger scale should be conducted to confirm our findings.

## Figures and Tables

**Table 1:** General clinical characteristics of the enrolled participants.

Variables	Mild-moderate	Severe OSAS	*P* value
	OSAS (*n* = 120)	(*n* = 71)	
Male	70 (58.3%)	55 (77.5%)	0.01
Smoking	67 (55.8%)	52 (73.2%)	0.02
Age (years)	54.1 ± 12	54.4 ± 10	0.86
BMI (kg/m^2^)	27.2 ± 1.7	27.0 ± 1.6	0.40
UA (*μ*mol/L)	413 ± 92.8	438 ± 95.9	0.08
Hcy (*μ*mol/L)	14.9 ± 6.0	17.4 ± 6.9	0.01
FBG (mmol/L)	5.7 ± 1.4	6.1 ± 2.2	0.15
TG (mmol/L)	1.8 ± 1.2	1.8 ± 0.9	0.78
TC (mmol/L)	4.4 ± 0.9	4.2 ± 0.9	0.44
LDL (mmol/L)	2.5 ± 0.7	2.4 ± 0.8	0.32
HDL (mmol/L)	1.4 ± 0.3	1.3 ± 0.4	0.48
*E*/*A* ratio	1.09 ± 0.2	1.08 ± 0.2	0.78
EF (%)	58.8 ± 6.7	58.6 ± 6.3	0.96
LVEDD (mm)	47.8 ± 4.2	48.4 ± 4.0	0.32

Values are expressed as the mean ± SD or %. Categorical values were compared by the *χ*^2^ test. Independent *t*-test or Wilcoxon's rank sum test for continuous values. Hcy: homocysteine; BMI: body mass index; UA: uric acid; FBG: fasting blood glucose; TG: triglyceride; TC: total cholesterol; LDL: low-density lipoprotein; HDL: high-density lipoprotein; EF: ejection fraction; LVEDD: left ventricular end-diastolic diameter; OSAS: obstructive sleep apnea syndrome.

**Table 2:** OSAS and HRV characteristics stratified by Hcy quartiles.

Variables	Hcy quartiles (*μ*mol/L)				
	Q1 (<11.3)	Q2 (11.3-14.3)	Q3 (14.4-18.3)	Q4 (>18.3)	*P*/*P* for trend
Severe/total	13/50	15/47	18/47	25/47	
OSAS, *N* (%)	(26%)	(31.9%)	(38.3%)	(53.2%)	0.04/0.01
SDANN	108 ± 33	103 ± 34	96 ± 29	95 ± 34	0.14/0.02
SDNN	60 ± 19	54 ± 19	54 ± 18	52 ± 20	0.26/0.07
pNN50	7 (3, 15)	8 (5, 16)	8 (2, 14)	5 (3, 10)	0.45/0.43
EF (%)	58.6 ± 6.6	58.5 ± 6.6	59.5 ± 6.4	58.4 ± 6.8	0.83/0.91
*E*/*A* ratio	1.06 ± 0.2	1.14 ± 0.2	1.09 ± 0.2	1.06 ± 0.2	0.18/0.64
LVEDD (mm)	47.6 ± 4.0	47.8 ± 4.0	48.6 ± 4.0	48.0 ± 4.5	0.64/0.41

Categorical values were compared by the *χ*^2^ test. Analysis of variance (ANOVA) or Wilcoxon's rank sum test for continuous values. SDNN: standard deviation of normal-to-normal intervals; SDANN: standard deviation of the averages of normal-to-normal intervals in all 5 min segments; pNN50: NN50 count divided by the total number of all normal-to-normal intervals.

**Table 3:** Relations of plasma Hcy to HRV and myocardium parameters.

Variables	Hcy			
	Crude		Adjust	
	*r*	*P*	*r*	*P*
SDANN	-0.17	0.02	-0.15	0.04
SDNN	-0.15	0.03	-0.10	0.16
pNN50	-0.08	0.27	-0.06	0.40
EF (%)	0.02	0.81	0.03	0.67
*E*/*A* ratio	-0.04	0.54	-0.12	0.09
LVEDD (mm)	0.05	0.49	0.03	0.72

Spearman's correlation analysis. Adjusted: age, sex, body mass index, blood pressure, triglyceride, total cholesterol, and severity of obstructive sleep apnea syndrome.

**Table 4:** The correlations between the HRV parameters and myocardium parameters.

Variables	SDANN		SDNN		pNN50	
	*r*	*P*	*r*	*P*	*r*	*P*
EF (%)	-0.04	0.58	-0.06	0.40	0.02	0.72
*E*/*A* ratio	-0.02	0.77	0.02	0.78	0.07	0.35
LVEDD (mm)	-0.02	0.76	0.06	0.37	0.09	0.18

Spearman's correlation analysis.

## Data Availability

The data used to support the findings of this study are available from the corresponding author upon request.
